# Efficacy and safety of linezolid compared with other treatments for skin and soft tissue infections: a meta-analysis

**DOI:** 10.1042/BSR20171125

**Published:** 2018-02-13

**Authors:** Yan Li, Wei Xu

**Affiliations:** Department of Dermatology, Beijing Friendship Hospital, Capital Medical University, Beijing 100050, China

**Keywords:** Linezolid, Meta-analysis, Skin and soft-tissue infections

## Abstract

Linezolid with other treatments for skin and soft tissue infections (SSTIs) has been evaluated in several studies. However, the conclusions remain controversial. By searching PubMed, EMBASE, and Cochrane library databases, we conducted a meta-analysis to evaluate linezolid and other treatments for skin and soft tissue infections. The study was summarized, and the risk ratio (RR) and its 95% confidence interval (CI) were calculated. Eleven related articles were included in the meta-analysis. Our results revealed that linezolid was associated with a significantly better clinical (RR = 1.09, 95% CI: 1.02–1.16, *P*_heterogeneity_ = 0.326, *I^2^* = 13.0%) and microbiological cure rates (RR = 1.08, 95% CI: 1.01–1.16, *P*_heterogeneity_ = 0.089, *I^2^* = 41.7%) when comparing with vancomycin. There was no significant difference in the incidence of anemia, nausea, and mortality; however, the incidence of vomiting, diarrhea, and thrombocytopenia in patients treated with linezolid is significantly higher than that with other treatments. Our study confirmed that linezolid seems to be more effective than vancomycin for treating people with SSTIs. It is recommended that linezolid be monitored for thrombocytopenia, vomiting, and diarrhea. Further studies with larger dataset and well-designed models are required to validate our findings.

## Introduction

Skin and soft tissue infections (SSTIs) represent one of the most common reasons for referral of emergency department (ED) and the most common cause of infection in the hospital. A recent strong growth trends in the incidence are mainly due to the expansion of the aging population with comorbidities and the emergence of community-acquired methicillin-resistant Staphylococcus aureus (CA-MRSA) [1,2]. In the United States, according to the national hospital ambulatory care survey database, visits for uncomplicated SSTIs (uSSTIs) almost doubled, between 1993 and 2005 (from 1.35 to 2.98%, *P*<0.001) [3]. In addition, the SSTIs hospitalization in the United States increased 30% between 2000 and 2004, mainly because superficial infection in adults is less than 65 years of age [4].

Linezolid was thought to be studying in SSTI because of activity against enterococcus, staphylococcus, and other gram-positive bacteria, including methicillin and vancomycin-resistant bacteria [5]. Linezolid is approved by the US Food and Drug Administration (FDA) for oral and intravenous injection of MRSA inf ection. Clinical efficacy and safety studies have been published by comparing linezolid with vancomycin in patients with severe infection, such as complex skin and skin structure infections (cSSSIs) [6], MRSA infection [7], febrile neutropenia [8], and hospital-acquired pneumonia [9,10], including the subsets with bacteremia [11].

Several studies have compared linezolid with other treatments for skin and soft tissue infections and no consistent outcomes are reported [12–22]. Recently, a systematic review and meta-analysis identified nine RCTs, which included a total of 3144 patients with skin and soft tissue infections, to assess the effect of linezolid and vancomycin for treating patients with SSTIs [23]. However, this meta-analysis only focused on linezolid and vancomycin, and several RCTs have been published on linezolid compared with new treatments for SSTIs. To provide a comprehensive assessment of efficacy and safety of linezolid compared with other treatments for skin and soft tissue infections, we performed a meta-analysis of published studies.

## Materials and methods

### Search strategy

We searched for relevant studies up to December 2016 through the PubMed, Embase, and Cochrane library databases with the following terms and their combinations: “linezolid”, “antibiotics” and “skin and soft-tissue infections”. All scanned abstracts, studies, and citations were reviewed. Moreover, references of the retrieved manuscripts were also manually cross-searched for further relevant publications.

### Selection criteria

The inclusion criteria included: (1) the study compared linezolid and other treatments; (2) the study had a randomized controlled trials design; (3) all participants in the present study were patients with SSTIs; 4) studies should report at least one of the outcomes with clinical cure, microbiological cure, anemia, nausea, vomiting, diarrhea, thrombocytopenia, and mortality; (5) the study provides risk ratios (RRs) with 95% confidence intervals (CIs) or enable calculation of these statistics from the data presented. The exclusion criteria included: (1) the studies which used the same population or overlapping database; (2) The studies of cell or animal models.

### Data extraction

All the available data were extracted from each study by two investigators independently according to the inclusion criteria listed above. The following data were collected from each study: first author name, publication year, country where the research was performed, number of patients, mean age, intervention method, treatment duration, and outcomes assessed.

### Statistical analysis

We calculated the risk ratio (RR) and 95% confidence intervals for dichotomous data. Data were combined according to random effects (DerSimonian and Laird’s method) or fixed effects model, depending on the significance of the *I^2^* statistic (*P*<0.1 and/or *I^2^*>50%). If the heterogeneity was significant, random effects model was used; otherwise the fixed effects model was used. In the sensitivity analysis, the influence of each study on the summary effect was analyzed by dropping one study at a time. Publication bias was evaluated by visual inspection of symmetry of Begg’s funnel plot and assessment of Egger’s test (*P*<0.05 was regarded as representative of statistical significance). Statistical analyses were done in STATA software, version 12.0 (STATA Corp., College Station, TX, U.S.A.), and all tests were two-sided.

## Results

### Characteristics of the studies

**Figure 1:**
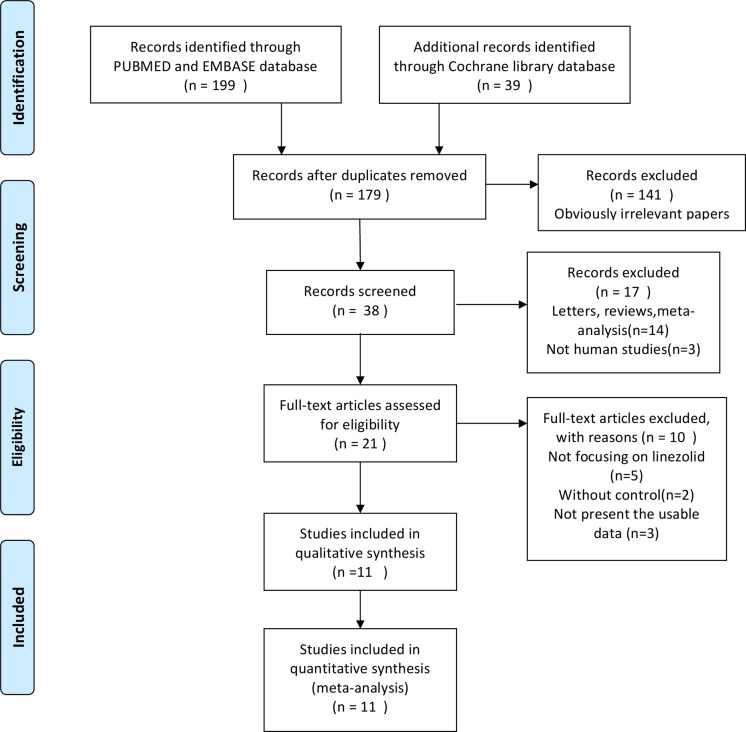
Flow diagram of studies identification. Flow diagram of studies identification.

There are 238 papers related to search terms. Subsequently, 200 unrelated articles were excluded. The remaining articles were systematically evaluated, and a total of 21 articles met the full text. After reading the full text, 10 articles were considered inappropriate and were excluded, and a qualitative analysis of the 11 articles was identified. Finally, 11 studies were included in the current meta-analysis. Exclude the research and choice flow chart is shown in Figure 1. The main features of the eligible study are shown in Table 1.

**Table 1:** Characteristics of randomized controlled trials included in this meta-analysis.

Authors/Year of publication	Mean age, years	Intervention		Treatment duration	Outcomes assessed
		Linezolid	Control		
Stevens/2002	Linezolid 63.9(16.1) Control 59.8(20.2)	600 mg IV twice daily, *N*=240	Vancomycin, 1 g IV twice daily, *N*=220	7–14 days	Clinical cure, microbiological cure
Yogev/2003	Linezolid 3.48(3.21) Control 3.03(2.87)	10 mg/kg IV every 8 h, *N*=80	Vancomycin, 10–15 mg/kg IV every 6–24 h, *N*=40	7–14 days	Clinical cure, microbiological cure, anemia, diarrhea, thrombocytopenia, mortality
Sharpe/2005	NA	600 mg orally every 12 h, *N*=30	Vancomycin, 1 g IV every 12 h, *N*=30	10 days	Clinical cure, microbiological cure
Weigelt/2005	Linezolid 52(18) Control 52(18)	600 mg every 12 h, IV or oral, *N*=592	Vancomycin, 1 g IV every 12 h, *N*=588	4–21 days	Clinical cure, microbiological cure, anemia, nausea, vomiting, diarrhea, thrombocytopenia, mortality
Jaksic/2006	Linezolid 47.2(15) Control 48.1(15.7)	600 mg IV every 12 h, *N*=304	Vancomycin, 1 g IV every 12 h, *N*=301	10–28 days	Clinical cure, microbiological cure
Kohno/2007	Linezolid 68.4(16.4) Control 67.5(16.3)	600 mg IV every 12 h, *N*=100	Vancomycin, 1 g IV every 12 h, *N*=51	7–28 days	Clinical cure, microbiological cure
Lin/2008	Linezolid 56.3(16.7) Control 59.6(13.3)	600 mg IV every 12 h, *N*=71	Vancomycin, 1 g IV every 12 h, *N*=71	7–21 days	Clinical cure, microbiological cure
Wilcox/2009	Linezolid 53.7(18.1) Control 53.8(17.6)	600 mg IV every 12 h, *N*=363	Vancomycin, 1 g IV every 12 h, *N*=363	7–28 days	Clinical cure, microbiological cure
Itani/2010	Linezolid 49.7(18–93) Control 49.4(18–99)	600 mg IV linezolid every 12 h, *N* = 537	Vancomycin, IV 15 mg/kg every 12 h, *N* = 515	7–14 days	Clinical cure, microbiological cure, nausea, vomiting, diarrhea
Noel/2012	NA	600 mg IV linezolid every 12 h, *N*=116	Omadacycline, 100 mg every 24 h, *N*=118	10–17 days	Clinical cure, microbiological cure, nausea, vomiting, diarrhea, mortality
Moran/2014	Linezolid 46(15–89) Control 46(17–86)	600 mg twice daily, *N*=334	Tedizolid, 200 mg once daily, *N*=332	7–14 days	Clinical cure, microbiological cure, nausea, vomiting, diarrhea, mortality

Abbreviations: IV, intravenous; NA, not available.

### Quantitative synthesis

**Figure 2:**
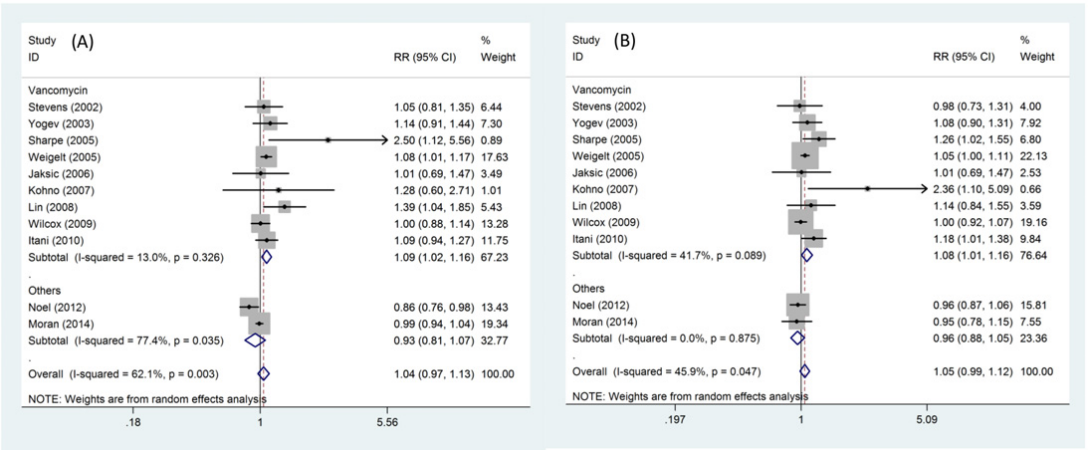
Efficacy outcomes in randomized controlled trials of linezolid versus other treatments. (**A**) clinical cure; (**B**) microbiological cure.

The 11 studies provided outcomes regarding the clinical cure rate in patients who received linezolid and other treatments, and were included in the meta-analysis. There was evidence of heterogeneity among the 11 studies; therefore, a random-effects model of analysis was used. There was no significant difference in the clinical cure (RR = 1.04, 95% CI: 0.97–1.13, *P*_heterogeneity_ = 0.003, *I^2^*=62.1%), as shown in Figure 2A. However, the pooled difference indicated that patients who received linezolid had significantly increased in the clinical cure compared with patients who received vancomycin (RR = 1.09, 95% CI: 1.02–1.16, *P*_heterogeneity_ = 0.326, *I^2^*=13.0%) (Figure 2 A).

The 11 studies provided outcomes regarding the microbiological cure rate in patients who received linezolid and other treatments, and were included in the meta-analysis. There was evidence of heterogeneity among the 11 studies; therefore, a random-effects model of analysis was used. There was no significant difference in the microbiological cure (RR = 1.05, 95% CI: 0.99–1.12, *P*_heterogeneity_ = 0.047, *I^2^*=45.9%), as shown in Figure 2B. However, the pooled difference indicated that patients who received linezolid had significantly increased in the microbiological cure compared with patients who received vancomycin (RR = 1.08, 95% CI: 1.01–1.16, *P*_heterogeneity_ = 0.089, *I^2^*=41.7%) (Figure 2B).

**Figure 3:**
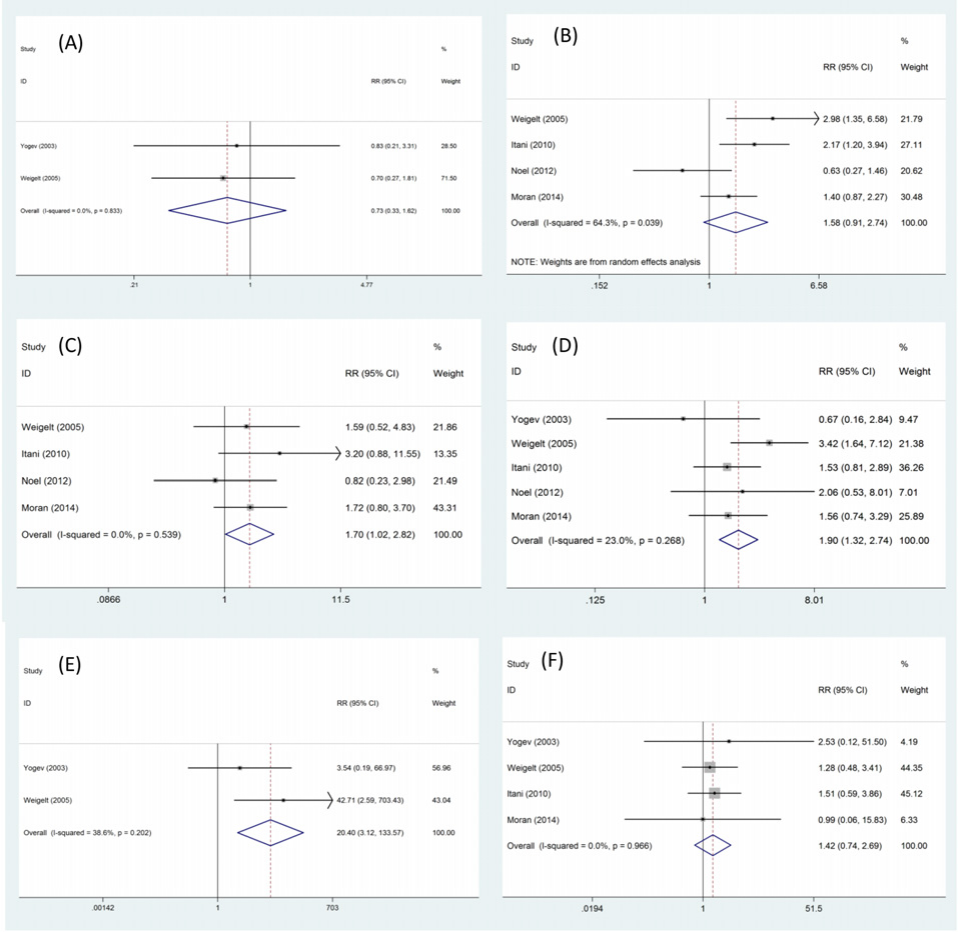
Adverse effects in randomized controlled trials of linezolid versus other treatments. (**A**) anemia; (**B**) nausea; (**C**) vomiting; (**D**) diarrhea; (**E**) thrombocytopenia; (**F**) mortality.

The five studies were included in the meta-analysis of adverse events:

Anemia: This outcome was reported in two trials, all comparing linezolid to vancomycin. There was no heterogeneity between the study (*P*=0.833, *I^2^*=0%), the fixed effect model was used. There was no significant difference in the incidence of anemia (RR = 0.73, 95% CI: 0.33–1.62), as shown in Figure 3A.

Nausea: This outcome was reported in four trials. There was significant heterogeneity between the study (*P*=0.039, *I^2^*=64.3%), the random effect model was used. There was no significant difference in the incidence of nausea (RR = 1.58, 95% CI: 0.91–2.74), as shown in Figure 3B.

Vomiting: This outcome was reported in four trials. There was no heterogeneity between the study (*P*=0.539, *I^2^*=0%), the fixed effect model was used. There was significant difference in the incidence of vomiting (RR = 1.70, 95% CI: 1.02–2.82), as shown in Figure 3C.

Diarrhea: This outcome was reported in five trials. There was no heterogeneity between the study (*P*=0.268, *I^2^*=23%), the fixed effect model was used. However, there was significant difference in the incidence of diarrhea (RR = 1.90, 95% CI: 1.32–2.74), as shown in Figure 3D.

Thrombocytopenia: This outcome was reported in two trials, all comparing linezolid to vancomycin. There was no heterogeneity between the study (*P*=0.202, *I^2^*=38.6%), the fixed effect model was used. However, there was significant difference in the incidence of thrombocytopenia (RR = 20.4, 95% CI: 3.12–133.57), as shown in Figure 3E.

Mortality: This outcome was reported in four trials. There was no heterogeneity between the study (*P*=0.966, *I^2^*=0%), the fixed effect model was used. There was no significant difference in the incidence of mortality (RR = 1.42, 95% CI: 0.74–2.69), as shown in Figure 3F.

### Sensitivity analysis

**Figure 4:**
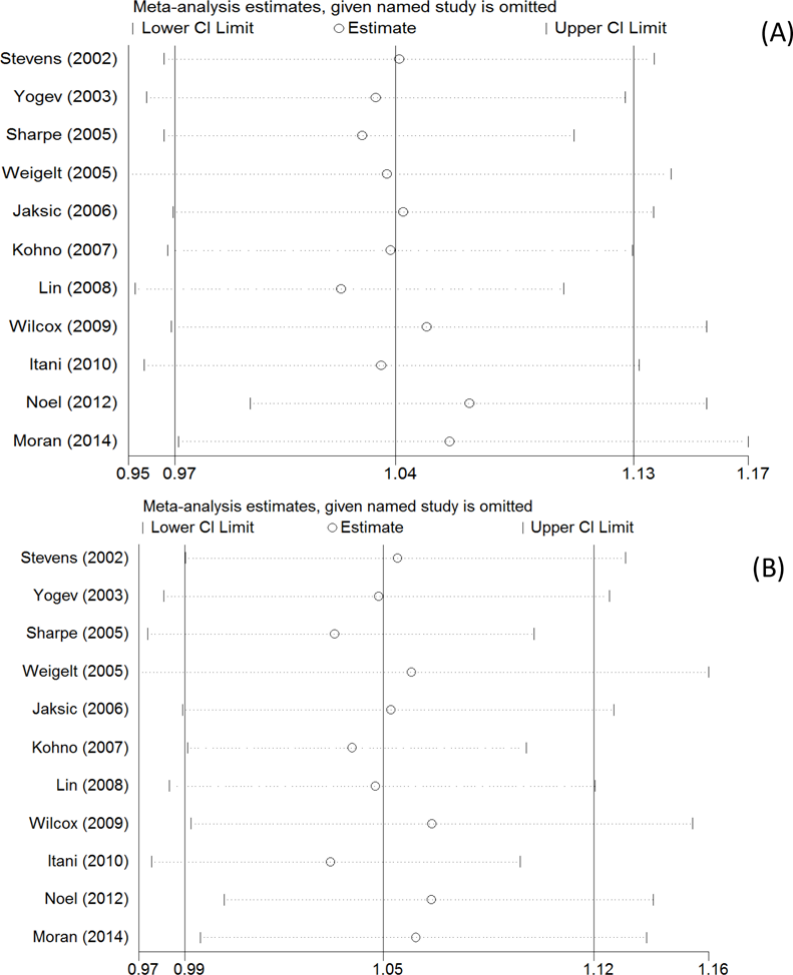
Sensitivity analysis for efficacy outcomes in randomized controlled trials of linezolid versus other treatments. (**A**) clinical cure; (**B**) microbiological cure.

We performed sensitivity analyses to assess the stability of the results by sequential removing each study. Any single study was removed, while the overall statistical results do not change (Figure 4), indicating that the results of the present study are statistically robust.

### Publication bias

**Figure 5:**
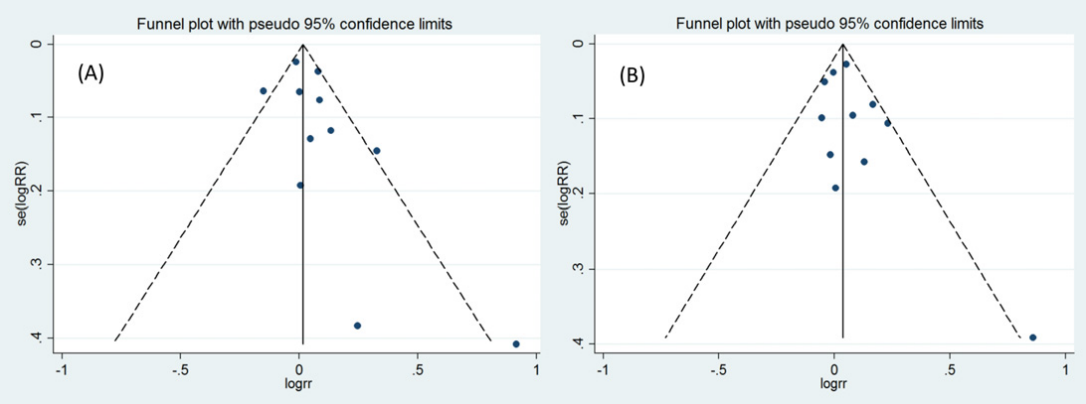
Begg’s funnel plot for publication bias test. Each point represents a separate study for the indicated association (**A**) clinical cure; (**B**) microbiological cure.

Finally, the Egger’s regression test showed no evidence of asymmetrical distribution in the funnel plot in the clinical cure (Begg’s test, *P*=0.119; Egger’s test, *P*=0.140) and the microbiological cure (Begg’s test, *P*=0.276; Egger’s test, *P*=0.234) (Figure 5).

## Discussion

Soft tissue infections, now known as acute bacterial skin and skin structure infections (ABSSSIs) are common in every medical profession and are encountered by everyone at certain times. ABSSSIs inflammatory microbial invasion of the epidermis, dermis, subcutaneous tissue, manifested as heat, various combinations, redness, swelling, and pain. Clinical management of ABSSSI is combined with surgical realization, support, and antimicrobial therapy [24,25]. New antibiotics are necessary because antibiotic resistance is a major global health hazard. If there is no new antibiotic, the further development of medical technology and the future of medical care may be at risk unless a global antibiotic resistance solution is found. Multiple drug-resistant bacteria are rapidly becoming ubiquitous, selected by uncontrolled antibiotic use and spread by poor infection prevention and public sanitation, and are quietly colonizing the global population. Antibiotics are a limited resource that saves lives in sepsis [26]. Their overuse is limited by the diagnosis, which means that the antibiotic is given to the patient who has no infection or viral infection [27]. The solution to this major problem seems simple but difficult to achieve [28,29].

The results of our meta-analysis showed linezolid was associated with a significantly better clinical (RR = 1.09, 95% CI: 1.02–1.16, *P*_heterogeneity_ = 0.326, *I^2^*=13.0%) and microbiological cure rate (RR = 1.08, 95% CI: 1.01–1.16, *P*_heterogeneity_ = 0.089, *I^2^*=41.7%) when comparing with vancomycin, implying that linezolid seems to be more effective than vancomycin for treating people with SSTIs. Methicillin resistant Staphylococcus aureus (MRSA) infection is associated with an increase in morbidity and mortality, largely related to the increased length of stay (LOS) associated with inpatient treatment of the infection [30,31]. Combined with the high cost of treatment, an increasing prevalence of MRSA in hospitals and communities presents challenges to healthcare systems [32,33]. Intravenous vancomycin is the first choice for the treatment of MRSA infection, but the emergence of resistant strains of Staphylococcus aureus in the clinical efficacy of this agent against MRSA [34,35]. Linezolid, an agent in the oxazolidinone class of antimicrobials, has clinical efficacy and toxicity profiles against MRSA similar to those of vancomycin and is 100% bioavailable orally. Linezolid, therefore, is an effective alternative to oral vancomycin in the treatment of MRSA infection in hospital and community settings.

The efficacy and safety of linezolid have been investigated by previous meta-analyses. As far as we know, this meta-analysis is the largest one to evaluate the efficacy and safety profile of linezolid up to now, which involved 5396 SSTIs patients from 11 RCTs. Recently, Yue et al. performed a meta-analysis about the efficacy and safety of linezolid in SSTIs patients [23]. Compared with Yue’s work, we identified more eligible studies involved more SSTIs patients and performed a detailed analysis, while Yue’s study only focused on linezolid and vancomycin.

At the same time, some limitations of this meta-analysis should be noted: first, our study may be compromised by extracting raw data from including research. Second, the dose associated with vancomycin is still difficult, and the more aggressive recommended dose has been established [37]. Vancomycin dose adjusted drug-based levels were not delineated in the study and may affect treatment outcome of MRSA cSSTI. A third of a potential limitation is that language can also introduce bias. Specifically, we only choose English to exclude other qualified researchers. Fourth, most of the RCTs included in the study were sponsored by the pharmaceutical companies that produce antimicrobials, and studies with the largest weight on results were the pivotal trials. Finally, only two studies met the inclusion criteria in some analysis, making it difficult to control any significant heterogeneity if it exists.

In conclusion, despite the limitations of this meta-analysis, our study confirmed that linezolid seems to be more effective than vancomycin for treating people with SSTIs. It is recommended that linezolid be monitored for thrombocytopenia, vomiting, and diarrhea. Further studies with larger dataset and well-designed models are required to validate our findings.

## Abbreviations

ABSSSI: acute bacterial skin and skin structure infection
CA-MRSA: community-acquired methicillin-resistant Staphylococcus aureus
CI: confidence interval
cSSSI: complex skin and skin structure infection
ED: emergency department
FDA: Food and Drug Administration
LOS: length of stay
MRSA: Methicillin resistant Staphylococcus aureus
RR: risk ratio
SSTI: skin and soft tissue infection
uSSTI: uncomplicated SSTI
